# A Treatise on Percussion and Auscultation of the Heart in the Healthy and Diseased States

**Published:** 1846-07

**Authors:** 


Art. IX.—*Percussion und Auscultation des Herzens im gesunden und kranken Zustande.* Von Liberal Günzburg, m.d. Zweite Auflagen. —*Wien,* 1844.

*A Treatise on Percussion and Auscultation of the Heart in the Healthy and Diseased States.* By Liberal Günzburg, m.d. Second edition. —*Vienna,* 1844. 8vo, pp. 179.

Although the soundness of the novel notions which Skoda has put forth concerning the mode of production (and, indeed, in some cases, the pathologiccal signification) of various phenomena of auscultation and percussion, may very fairly be questioned, and though those notions have not been admitted, nor are likely to be admitted, by any but his immediate pupils and those whom local influences may be expected to convert, yet it cannot be denied that his writings have been of very material service. In truth, the Germans now feel a sort of property in auscultation, and are stimulated to make its mysteries more and more completely their own; whereas, before the advent of Skoda, their ignorance of and indifference generally to the subject, were matters of remark on the part of all who visited their schools. There is now a glut of manuals, treatises, &c. of auscultation in the German market, even greater than in our own or in that of France.

It is not sufficiently known (ingenious devices have in this country been employed to throw the fact into the shade) that Dr. Carswell was the real proposer of the theory of the second sound of the heart, ascribing the phenomena to flapping of the semi-lunar valves. It is, on the other hand, well known that, after that theory had been put forward by Carswell, Billing, and Rouanet, several persons refused to accept it; Dr. Williams, for example, (in 1833) held that the second sound was produced by tension of the parietes of the ventricles. Such being the facts, it scarcely surprised us to find, in this manual, no mention of Dr. Carswell's name in connexion with the point. Skoda's fantastic theory of the cardiac sounds (as fantastic in its way as any product of any German brain) is not accepted by the author.

Dr. Günsburg makes no reference in his description of the origin of pericarditis to the statement of M. Gendrin to the effect that bulging of the pericardial region occurs previously to the production of fluid effusion in the pericardium. We do not quarrel with him for this; we believe the appearance in question to have existed very much in the "mind's eye" of M. Gendrin, and nowhere else. The discovery was in every way worthy of that remarkable acuteness of vision whereof M. Gendrin gave such marvellous proof in times of yore by "*seeing*" (this was the word,) the *red corpuscles of the blood* converted into *pus-corpuscles within* the capillary vessels, and transuding thence ready-formed into the circumambient textures!

Besides the exposition of the physical characters of diseases of the heart, this work contains full tabular views of pulmonary and cardiac diseases "in their diagnostic and anatomico-pathological relations, after Skoda and Rokitansky, with an appendix on their treatment." The title of this portion of the work gives a fair insight into its general character and pretensions: it is, in truth, got up "after" the labours of others,—a simple and correct compilation, but nothing more.

